# Pharmacokinetics and Genetic Factors of Atorvastatin in Healthy Korean Subjects

**DOI:** 10.3389/fgene.2022.836970

**Published:** 2022-05-19

**Authors:** Serim Kim, Jong Do Seo, Yeo-Min Yun, Hanah Kim, Tae-Eun Kim, Taeheon Lee, Tae-Rim Lee, Jun Hyung Lee, Eun-Hae Cho, Chang-Seok Ki

**Affiliations:** ^1^ Department of Laboratory Medicine, Shinwon Medical Foundation, Gwangmyeong-si, South Korea; ^2^ Department of Laboratory Medicine, Konkuk University Medical Center, Seoul, South Korea; ^3^ Department of Laboratory Medicine, Konkuk University School of Medicine, Seoul, South Korea; ^4^ Department of Clinical Pharmacology, Konkuk University Medical Center, Seoul, South Korea; ^5^ GC Genome, Yongin-si, South Korea; ^6^ Department of Laboratory Medicine, GC Labs, Yongin-si, South Korea

**Keywords:** atorvastatin, pharmacokinetic parameter, pharmacogenetics, next-generation sequencing, liquid chromatography-tandem mass spectrometry

## Abstract

**Background:** Statins are the most popular agents for the primary and secondary prevention of cardiovascular disease; however, the pharmacokinetic parameters and associated genetic factors in the Korean population have not been fully elucidated. This study explored the pharmacokinetic properties of atorvastatin and the association between genetic variations and atorvastatin pharmacokinetics in healthy Korean subjects.

**Methods:** Atorvastatin (80 mg) was administered to 35 healthy Korean volunteers. Plasma levels of atorvastatin and its metabolites were measured sequentially using liquid chromatography-tandem mass spectrometry from 0 to 24 h after atorvastatin administration. Customized next-generation sequencing analysis was performed covering all coding exons of 15 genes, as well as 46 single-nucleotide variants in 29 genes related to statin pharmacokinetics.

**Results:** The mean area under the concentration-time (AUC) and C_max_ (maximum peak concentration) were 269.0 ng/ml∙h and 84.3 ng/ml, respectively, which were approximately two times higher than those reported in Caucasians. Genetic analysis revealed that eight genetic variants in *ABCB1, ABCG2, APOA5, CETP*, and *CYP7A1* contributed to the AUC of atorvastatin. The atorvastatin AUC_0–24 h_ prediction model was developed based on age and eight genetic variants using multivariate linear regression (adjusted *R* ^2^ = 0.878, *p* < 0.0001).

**Conclusion:** This study shows that the pharmacokinetic properties of atorvastatin in Koreans are different from those in Caucasians and that atorvastatin AUC_0–24 h_ could be predicted based on age and eight genetic variants of *ABCB1, ABCG2, APOA5, CETP,* and *CYP7A1*.

## Introduction

Statins are used as a first-line treatment for the primary prevention of atherosclerotic cardiovascular disease (ASCVD) in patients with elevated low-density lipoprotein cholesterol (LDL-C) levels, those with diabetes mellitus between the age of 40–75 years, and those who are at sufficient ASCVD risk as assessed by clinician-patient risk discussion (Arnett et al., 2019). Atorvastatin and rosuvastatin are prescribed as drugs of choice for high-or medium-intensity statin therapy (Adams et al., 2015).

Adverse events associated with statin use are a major cause of noncompliance; the most common being statin myositis and statin-associated muscle symptoms (SAMS) (Law and Rudnicka, 2006). Although the mechanisms of SAMS are not completely understood, it is considered that the risk of myopathy is dose-related and dependent on systemic drug exposure (Holbrook et al., 2011). However, not all patients receiving the same dose experience adverse events or have the same LDL-C lowering effect, implying that genetic variants affecting statin pharmacokinetics (PK) may influence the incidence and severity of statin adverse events. The Clinical Pharmacogenetics Implementation Consortium (CPIC) prescribing guidelines for simvastatin based on the *SLCO1B1* variant is a commonly cited example of formal guidance regarding pharmacogenetics related to statin toxicity (Ramsey et al., 2014).

Reports on the effects of genetic variations on statin pharmacokinetics have been inconsistently replicated, and there are limited data available in Asian populations (Gandelman et al., 2012). Moreover, there are many variations in metabolic processes according to statin type, frequency of genetic variants, and responses to statins according to ethnic background (Liao, 2007). Results of the HPS2-THRIVE study evaluating the effect of extended release niacin plus laropiprant (ERN/LRPT) on statin adverse events indicated that the incidence rate of myopathy and increased liver enzymes in the Chinese population was approximately 3 times higher than that in Europeans with the same statin dose (simvastatin 40 mg daily) and it can increased up to 10 times higher with additional ERN/LRPT regimen (Group, 2013).

The aim of our study was to explore the PK properties of atorvastatin, to investigate the association between atorvastatin PK and genetic variants during the early phase of PK after oral administration of atorvastatin in healthy Korean subjects, and to establish a PK prediction model based on genetics.

## Materials and Methods

### Study Population and Sample Collection

This study was approved by the Institutional Review Board of Konkuk University Medical Center (KUH1200102) and the Korean Ministry of Food and Drug Safety (KMFDS). Thirty-five healthy Korean volunteers between the ages of 20 and 55 were enrolled, with written informed consent from each participant. All the subjects were confirmed to be eligible for the study, not having any current or pre-existing medical condition, not taking any medications, and showing no abnormal findings on the laboratory tests. Medical record review and routine check for admission of inpatients, including physical examination, vital signs, electrocardiogram, and laboratory tests, were performed. The subjects received a single oral dose of 80 mg atorvastatin calcium at 8:30 a.m. in an overnight fasting state. After 4 h of additional fasting, lunch and dinner were permitted. Venous blood was collected via an intravenous catheter prior to drug administration (0 h) and at 0.25, 0.5, 0.75, 1, 1.5, 2, 3, 4, 6, 8, 11, and 24 h after dosing for pharmacokinetic analysis. After collection, all samples were immediately centrifuged, aliquoted, and stored frozen. Genetic analysis was performed using the first blood sample (0 h).

### Measurement of Drug Concentrations and Biochemical Variables

The plasma concentrations of atorvastatin and its two active metabolites [para-hydroxy (p-OH) and ortho-hydroxy (o-OH) atorvastatin] were measured by liquid chromatography-tandem mass spectrometry (LC-MS/MS) using a 5500 QTRAP mass spectrometer (Sciex, Framingham, MA, United States) at Green Cross Laboratories (Yongin-si, Gyeonggi-do, South Korea). Standard compounds of atorvastatin, p-OH atorvastatin, o-OH atorvastatin, and deuterium-labeled atorvastatin (atorvastatin-d5) were purchased from TLC Pharmaceutical Standards Ltd. (Newmarket, Ontario, Canada). LC-MS/MS analysis was performed with the protocol that authors have presented as poster at the 11th Annual North American Conference of The Association for Mass Spectrometry: Applications to the Clinical Lab (Kim et al., 2019), by using atorvastatin-d5 (1 μg/ml in 50% acetonitrile) as an internal standard (IS).

The actual concentration-time profile was plotted, and the PK parameters were determined using non-compartmental analyses by BA-Calc 2007 (Ministry of Food and Drug Safety, Osong, South Korea) and PKSolver (Zhang et al., 2010). The maximum plasma concentration (C_max_) and time to achieve C_max_ (T_max_) were determined directly from the plasma concentration. The rate constant was calculated by performing a linear regression of the logarithmic concentration-time curve in the terminal phase. The area under the plasma concentration curve from 0 h to infinity (AUC_∞_) was calculated using a combination of the linear trapezoidal method and extrapolation to infinity by the elimination rate constant.

### Genetic Analysis

As shown in Supplementary Table S1, 15 genes and 46 non-exon single nucleotide polymorphisms (SNPs) in 29 genes were selected for genetic analysis based on associations with the absorption, distribution, metabolism, and elimination (ADME) of statins and their effects on lipid metabolism, as compiled by the PharmGKB database and previous studies (Mangravite et al., 2006; Wang et al., 2011; Canestaro et al., 2014; Leusink et al., 2016; Maxwell et al., 2017). The SNP of TCF7L2 is also added to this panel, which is known to be associated with the risk of diabetes mellitus (DM) as a side effect of statins (Chen et al., 2018). Genomic DNA was extracted from EDTA whole blood using a Chemagen DNA Blood Kit (PerkinElmer, Baesweiler, Germany). A custom panel (Integrated DNA Technologies, Coralville, Iowa, United States) was used for library preparation, and sequencing was performed on an Illumina MiSeqDX (Illumina Inc., San Diego, CA, Unites States), generating 2 × 150 bp paired-end reads. Alignment of sequence reads, indexing of the reference genome (hg19), and variant calling with a pipeline were based on Genome Analysis Tool Kit (GATK) Best Practices (DePristo et al., 2011). Alignment was performed using Burrows-Wheeler Aligner-maximum exact matches (BWA-MEM), version 0.7.12 (http://bio-bwa.sourceforge.net), and duplicated reads were marked with Picard, version 1.138 (http://picard.sourceforge.net). Local alignment, base quality recalibration, and variant calling of each sample was performed using the HaplotypeCaller of GATK, version 3.5 (Broad Institute, Cambridge, MA, United States) (McKenna et al., 2010) in the -ERC GVCF mode. After generating GVCFs for all samples, joint genotyping was performed using GATK GenotypeGVCFs. The VCF cohort was converted to a PLINK format using PLINK v1.9. Variant annotation was performed using Variant Effect Predictor (VEP) (McLaren et al., 2016) and non-synonymous functional predictions (dbNSFP) (Liu et al., 2016).

### Statistical Analysis

Prior to conducting statistical analysis, quality control procedures were implemented on both genotype and phenotype data. SNPs with missing rates of >5%, minor allele frequencies of <0.01, or significant deviation from Hardy–Weinberg equilibrium (*p* < 0.00001) were removed. The removed SNPs were rs4987144, rs1985842, rs111564371, rs112568578, rs113889384, rs28371713, and rs111606937 in *CYP2D6*. As quantitative trait association analysis assumes that the phenotypes are normally distributed, a rank-based inverse normal transformation method was applied to the raw phenotype values according to a previous report (Goh and Yap, 2009). Linear regression analysis for each SNP was performed using PLINK v1.9 (http://pngu.mgh.harvard.edu/purcell/plink) (Purcell et al., 2007) after adjusting for age, sex, and body weight.

From the results of linear regression for each of 467 SNPs, 24 SNPs were used for variable selection with *p*-value ≤0.05. Stepwise regression was performed in order to develop Statin AUC prediction model. The regression was run in R version 4.0.5 using the stepAIC() function from the MASS library. Starting from a model that included all variables (24 SNPs and age), either deleted the variable that was least helpful to the standard statistic or added the variable that improved the standard statistic most, among the missing variables in the model. Then, the addition or deletion of these SNP variables had been repeated. As a result, eight of the 24 variants were selected with age as the essential variable, and the dosage information of all 35 samples was the same as 80 mg. We adjusted the formula using the dosage value/80 (Equation in Table 3). The genotypes were coded as 0, 1, or 2 (additive effect model). Statistical analyses were performed using R version 4.0.5.

## Results

### Participant Demographics

The baseline characteristics of the 35 participants are summarized in Table 1. The male to female ratio was 6:4, and the average age, height, and weight were 34.5 years (range, 22–52 years), 168.7 cm (range, 151.1–182.9 cm), and 68.6 kg (range, 52.5–90.7 kg), respectively. Mean plasma concentrations (mg/dl) were as follows: total cholesterol, 171.98; HDL-C, 50.30; LDL-C, 99.62; triglycerides, 103.70; ApoA-I, 135.41; ApoA-II, 29.91; ApoB, 82.17 7, and Lp(a),27.48 nmol/L.

**TABLE 1:** Characteristics of the study population (*n* = 35).

Variables	Mean (Range)
Age (years)	34.5 (22–52)
Gender, *n* (%) Male Female	21 (60.0) 14 (40.0)
Height (cm)	168.7 (151.1–182.9)
Weight (kg)	68.6 (52.5–90.7)
Baseline lipid level	—
Total cholesterol (mg/dl)	171.98 (122.70–245.10)
HDL-C (mg/dl)	50.30 (31.62–80.93)
LDL-C (mg/dl)	99.62 (58.60–168.69)
Triglycerides (mg/dl)	103.70 (22.70–242.40)
Lp(a) (nmol/L)	27.48 (3.50–261.10)
ApoA-I (mg/dl)	135.41 (104.00–189.40)
ApoA-II (mg/dl)	29.91 (19.60–43.90)
ApoB (mg/dl)	82.17 (50.30–128.40)

Abbreviations” HDL-C, high-density lipoprotein cholesterol; LDL-C, low-density lipoprotein cholesterol; Lp(a), lipoprotein(a); ApoA-I, apolipoprotein A-I; ApoA-II, apolipoprotein A-II; ApoB, apolipoprotein B.

### PK of Atorvastatin

The PK parameters of atorvastatin were as follows: the mean AUC_0–24 h_ was 269.0 ng∙h/mL; AUC_∞_ was 279.4 ng∙h/mL; C_max_ was 84.3 ng/ml; time to reach maximum plasma concentration (T_max_) was 1.4 h, and elimination half-life (T_1/2_) was 5.5 h (Table 2). PK parameters showed marked inter-individual variability, with a coefficient of variation ranging from 17.8 to 60.8%. The values of AUC_0–24 h_ and C_max_ obtained in this study were about two times higher than those obtained in a white population (269.0 vs. 146.8 ng∙h/mL and 84.3 vs. 34.8 ng/ml, respectively) (Gandelman et al., 2011).

**TABLE 2:** Comparison of pharmacokinetic parameters of atorvastatin and its metabolites between our study subjects and other reports.

	This study			Woo H et al^a^	Gandelman K et al.	Braeckman RA et al.	Birmingham BK et al. †		
—	Koreans (*n* = 35)	Male (*n* = 21)	Female (*n* = 14)	Korean young males (*n* = 50)	White (*n* = 73)	Mixed ethnicity (*n* = 30)	Chinese (*n* = 31)	Japanese (*n* = 30)	Caucasian (*n* = 30)
ATV administration dose (mg)	80	80	80	80	80	80	40	40	40
**ATV**	Mean (SD)	Mean (SD)	Mean (SD)	Mean (SD)	Mean (SD)	Mean (SD)	Gmean (95% CI)	Gmean (95% CI)	Gmean (95% CI)
AUC_0–24 h_ (ng ∙h/ml)	269.0 (122.2)	264.5 (90.9)	277.4 (162.2)	NR	146.8 (119.7)	179.8 (59.5)	111 (93.7–133)	123 (103–147)	72.7 (61.0–86.8)
AUC_∞_ (ng ∙h/ml)	279.4 (127.2)	275.7 (96.0)	284.4 (167.8)	172 (94.9)	NR	NR	NR	NR	NR
C_max_ (ng/ml)	84.3 (39.4)	82.1 (28.5)	87.7 (52.8)	36.2 (19.3)	34.8 (36.5)	52.7 (19.3)	21.8 (18.1–26.3)	23.3 (19.3–28.2)	13.6 (11.3–16.5)
T_max_ (h)	1.4 (0.8)	1.2 (0.7)	1.6 (0.8)	1.07 (0.89)	NR	1.00	0.55 (0.55–3.00)^a^	1.00 (0.50–5.02)^a^	0.76 (0.50–5.00)^a^
CL (L/h)	337.3 (133.8)	319.8 (98.2)	363.6 (175.3)	603 (310)	NR	NR	NR	NR	NR
T_1/2_ (h)	5.5 (1.0)	5.9 (1.0)	5.0 (0.8)	7.75 (2.56)	NR	NR	7.85 (39.3, CV%)	7.47 (51.4, CV%)	7.08 (45.9, CV%)
**p-OH ATV**	Mean (SD)	Mean (SD)	Mean (SD)	Mean (SD)	Mean (SD)	Mean (SD)	Gmean (CV%)	Gmean (CV%)	Gmean (CV%)
AUC_0–24 h_ (ng ∙h/ml)	29.2 (10.0)	28.7 (7.0)	29.8 (13.2)	NR	NR	36.7 (16.4)	14.1 (80.1)	14.6 (102)	5.71 (229)
C_max_ (ng/ml)	3.0 (1.7)	2.7 (1.5)	3.3 (2.0)	NR	NR	4.1 (2.2)	0.82 (67.0)	0.97 (72.6)	0.57 (59.2)
T_max_ (h)	3.1 (2.4)	2.9 (2.4)	3.5 (2.4)	NR	NR	1.50	NR	NR	NR
AUC_0-t_ metabolites/ parent ratio	0.1 (0.03)	0.1 (0.02)	0.1 (0.03)	NR	NR	NR	0.13 (55.2)	0.12 (64.9)	0.08 (146.1)
**o-OH ATV**	Mean (SD)	Mean (SD)	Mean (SD)	Mean (SD)	Mean (SD)	Mean (SD)	Gmean (CV%)	Gmean (CV%)	Gmean (CV%)
AUC_0–24 h_ (ng ∙h/ml)	243.7 (71.4)	240.9 (71.3)	247.7 (74.0)	NR	NR	213.1 (73.0)	172 (37.3)	170 (41.1)	135 (32.4)
C_max_ (ng/ml)	51.8 (20.3)	50.0 (21.0)	54.6 (19.6)	NR	NR	43.2 (18.8)	16.5 (48.6)	18.3 (52.3)	13.1 (46.0)
T_max_ (h)	1.9A (1.1)	1.9 (1.3)	1.9 (0.7)	NR	NR	1.25	NR	NR	NR
AUC_0-t_ metabolites/ parent ratio	1.0 (0.4)	1.0 (0.3)	1.1 (0.5)	NR	NR	NR	1.55 (34.9)	1.38 (32.4)	1.86 (33.0)

a The study subjects of Woo H et al. were young healthy men with an average age of 24 years. † Since the atorvastatin administration dose of Birmingham BK, et al. is half (40 mg) compared to other studies (80 mg), it is recommended to double the AUC, and Cmax data of Birmingham BK, et al. when comparing those of other studies.

Abbreviations: ATV, atorvastatin; p-OH ATV, para-hydroxy atorvastatin; o-OH ATV, ortho-hydroxy atorvastatin; SD, standard deviation; CV%, coefficient of variation; AUC0-24 h, area under the plasma concentration curve from hour 0 to observation, AUC∞, area under the plasma concentration curve from hour 0 to infinity; Cmax, maximum plasma drug concentration; Tmax, time to reach maximum plasma concentration following drug administration; CL, apparent total clearance of the drug from plasma after oral administration; T1/2, elimination half-life; NR, not reported.

**FIGURE 1:**
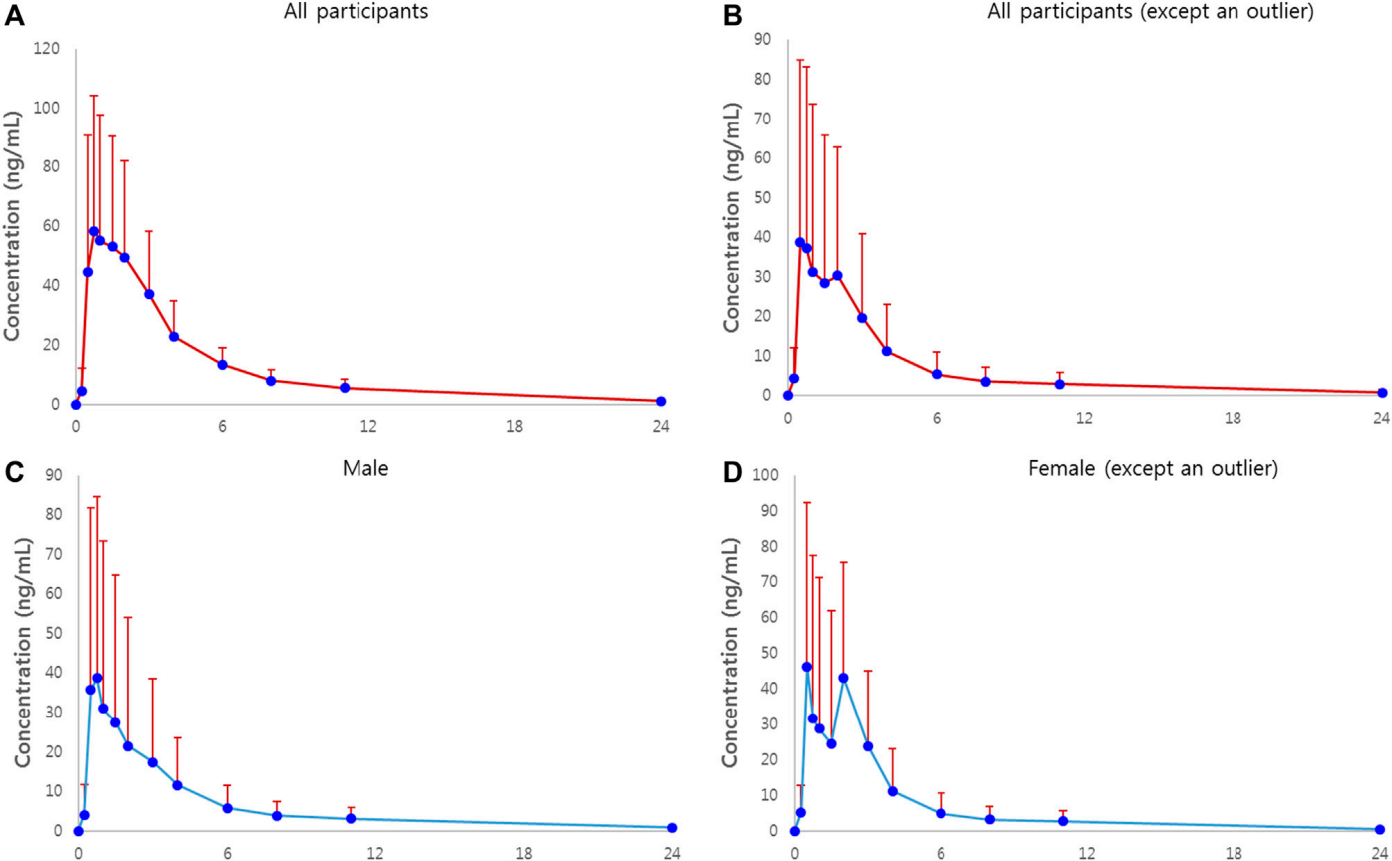
Plasma concentration-time plots of atorvastatin in healthy subjects receiving a single dose of 80 mg atorvastatin. **(A)** Plot of all participants. Solid circles represent mean concentrations of plasma atorvastatin, and bars represent standard deviations. **(B)** Plot of all participants, excluding an outlier of AUC_∞_ of atorvastatin. **(C)** Plot of all male participants. **(D)** Plot of all female participants, excluding an outlier of AUC_∞_ of atorvastatin.

The plasma concentration-time plots of atorvastatin after administration are shown as mean ± standard deviation (SD) values in Figure 1A. Figures 1B–D show the mean plasma concentration-time profile and the gender-differentiated profile after excluding one female outlier whose PK characteristics for atorvastatin were extremely high.

### Genetic Variants Associated With PK Parameters

A total of 467 variants were identified. Twenty-one genetic variants from seven genes were associated with PK parameters according to genotype (*p* < 0.01 in linear regression analysis; Supplementary Table S2). However, no genetic variants had a false discovery rate (FDR) *p*-value of <0.05.

In multivariate linear regression using stepwise selection to create a statin AUC prediction model, eight genetic variants of *ABCB1, ABCG2, APOA5, CETP,* and *CYP7A1* were selected, with age as the essential variable to predict atorvastatin AUC_0–24 h_ (adjusted *R* ^2^ = 0.878, *p* < 0.0001) (Table 3).

**TABLE 3:** Multivariate linear regression analysis for AUC_0–24 h_ prediction based on the genetic variants affecting the pharmacokinetics of atorvastatin.

Associated gene	Variables	Code of genotypes			Estimate (B)	Standard Error of estimate	t-value	*p*-value
		0	1	2				
*ABCB1*	rs2235029_C	A/A	A/C	C/C	−126.0	46.76	−2.70	0.012
*ABCB1*	rs3214119_T	TC/TC	TC/T	T/T	118.9	22.04	5.40	<0.001
*ABCB1*	rs1922242_T	A/A	A/T	T/T	60.0	14.65	4.09	<0.001
*ABCG2*	rs759701118_A	G/G	G/A	A/A	358.6	46.73	7.68	<0.001
*APOA5*	rs33984246_G	A/A	A/G	G/G	118.4	45.13	2.62	0.015
*APOA5*	rs3135507_T	C/C	C/T	T/T	−94.2	48.30	−1.95	0.062
*CETP*	rs12708974_T	C/C	C/T	T/T	−49.4	18.62	−2.65	0.014
*CYP7A1*	rs3808607_T	G/G	G/T	T/T	46.4	12.96	3.59	0.001
—	Age	—	—	—	2.7	0.83	3.31	0.003
—	(Intercept)	—	—	—	65.558	32.56	2.014	0.055
Multivariate linear regression analysis for ATV AUC_0-24 h_ prediction								
R^2^		0.911						
Adjusted R^2^		0.878						
F-statistic		28.3						
*p* value		<0.001						
Equation for atorvastatin AUC_0-24 h_								
Atorvastatin AUC_0–24 h_ = [65.558 + 2.737 × Age-49.444×rs12708974 (*CETP*) + 118.397 × rs33984246 (*APOA5*) + 46.449×rs3808607 (*CYP7A1*)−94.225 × rs3135507 (*APOA5*)−126.04 × rs2235029 (*ABCB1*) + 118.925 × rs3214119 (*ABCB1*) + 59.991 × rs1922242 (*ABCB1*) + 358.648 × r s759701118 (*ABCG2*)] × 80/dosage								

Eight genetic variants (*ABCB1* rs2235029_C, rs3214119_T, rs1922242_T; *ABCG2* rs759701118_A; *APOA5* rs33984246_G, rs3135507_T; *CETP*, rs12708974_T; *CYP7A1* rs3808607_T) and age showed significant association with the ATV AUC, in the multivariate linear regression analysis.

Two individuals had PK parameter values higher than twice the average values for the other patients: one in AUC∞ and Cmax of atorvastatin (Participant 16: 40 years old, woman), and the other in AUC0-24 h and Cmax of metabolites (Participant 11: 51 years old, man). Genetic variants identified only in these two individuals are shown in Supplementary Table S3, and their plasma drug concentration-time plots are shown in Supplementary Figure S1.

## Discussion

In this study, the PK properties of atorvastatin in healthy Korean subjects differed from those previously reported (Table 2). The average atorvastatin AUC_0–24 h_ and C_max_ in this study were relatively higher than those reported by Woo et al. for the Korean population, which may be associated with the relative decrease in atorvastatin elimination capability (relatively low CL/F of 56% compared to that of Woo et al.) due to differences in the composition of the study subjects (age range from 21 to 51 years, including women vs. young, healthy men with an average age of 24 years) (Woo et al., 2017). In the multivariate linear regression analysis to estimate atorvastatin AUC, old age showed a significant association with the increasing atorvastatin AUC (Table 3), and this finding agrees well with a previous study that revealed a decrease in ATV elimination with increasing age (Gibson et al., 1996). This previous study revealed the shorter half-life in women agree with present study, however it showed lower AUC in female contrary to our study.

In addition, the average AUC_0–24 h_ and C_max_ in this study were about two times higher than those reported in a white population (269.0 vs. 146.8 ng∙h/mL and 84.3 vs. 34.8 ng/ml, respectively) (Gandelman et al., 2011). This finding suggests that with a high-dose (80 mg) treatment, atorvastatin exposure is relatively higher in the Korean population than in the Western population. Previous studies have reported higher rosuvastatin exposure in Asian populations compared to that in Caucasians (Lee et al., 2005; Li et al., 2007). The AUC for Asians was approximately twice that of Caucasians with the same dose of rosuvastatin, suggesting that Asians are more sensitive to statins (Lee et al., 2005). A comparative study on the systemic exposure of rosuvastatin, atorvastatin, and simvastatin in Caucasian and Asian subjects indicated that not only rosuvastatin but also atorvastatin exposure is higher in the Asian population than in the Caucasian population (Birmingham et al., 2015). The geometric mean AUC from time zero to the last quantifiable concentration was 86% (90% confidence interval, 51–130%) and 55% (26–91%) higher for rosuvastatin in Chinese and Japanese subjects than in Caucasians, respectively, and 53% (25–88%) and 69% (37–108%) higher for atorvastatin (Birmingham et al., 2015). Furthermore, the genetic variants in *SLCO1B1* T521 > C or *ABCG2* C421 > A were associated with higher exposure to rosuvastatin, atorvastatin, and simvastatin acid (Birmingham et al., 2015). However, even in the absence of both these genetic variants, ethnic differences in exposure were still observed; the underlying mechanism is suggested to be related to increased statin absorption in Asians compared with Caucasians (Birmingham et al., 2015). The finding of relatively high AUC and C_max_ in this study is consistent with a previous report demonstrating higher statin exposure in Asian subjects compared to Caucasians, suggesting that increased statin exposure may be associated with the higher incidence rate of statin side effects in Asian populations than in Western populations.

We selected 15 genes and 46 non-exon SNPs in 29 genes for analysis. A total of 467 genetic variants were found in the 35 Korean subjects, and among them, 21 variants from seven genes were associated with PK parameters according to genotype (*p* < 0.01 in linear regression analysis; Supplementary Table S1). In multivariate linear regression using stepwise selection for the creation of a statin AUC prediction model, eight genetic variants of *ABCB1* (rs2235029_C, rs3214119_T, rs1922242_T), *ABCG2* (rs759701118_A), *APOA5* (rs33984246_G, rs3135507_T), *CETP* (rs12708974_T), and *CYP7A1* (rs3808607_T) and age were selected to predict atorvastatin AUC_0–24 h_ (adjusted R2 = 0.878, *p* < 0.0001) (Table 3).

In our study, genetic variants of *ABCB1* (rs2235029_C, rs3214119_T, rs1922242_T) and *ABCG2* (rs759701118_A) were significantly associated with the level of atorvastatin exposure (AUC) (Table 3). The efflux ATP-binding cassette transporters of ABCB1 and ABCG2 play a key role in the pharmacokinetics, safety, and lipid-lowering efficacy of statins (Niemi, 2010; Tomlinson et al., 2010; Chasman et al., 2012). ABCB1 transport protein is involved in hepato-biliary and renal-urinary transport of statins and metabolites. Functional genetic variants in *ABCB1* and *ABCG2* related to reduced activity of the transporters have been found to be significantly associated with increased statin exposure (Niemi, 2010) and affect the safety and lipid-lowering efficacy of statins (Hu et al., 2010; Chasman et al., 2012). The functional genetic variant of 421 C > A (rs2231142) in *ABCG2*, that reduces transporter activity has been found to be associated with increased systemic exposure and lipid-lowering effect of statins (Hu et al., 2010; Chasman et al., 2012). The functional genetic variant of 421 C > A (rs2231142) in *ABCG2* to reduce transporter activity was associated with increased systemic exposure and the lipid-lowering effect of statins (Hu et al., 2010; Tomlinson et al., 2010; Chasman et al., 2012). Genetic variants in *ABCG2* C421 > A are associated with higher exposure to atorvastatin in Chinese and Japanese subjects than in Caucasians (Birmingham et al., 2015). However, since an ethnic difference in exposure was still observed in the absence of the *ABCG2* 421 C > A variant, another genetic change could differentially impact the function of *ABCG2* between populations (Birmingham et al., 2015). Since *ABCB1* and *ABCG2* play an essential role in statin transport, the genetic variations of *ABCB1* and *ABCG2* may be related to differences in the degree of statin exposure between individuals or races.

Bile acid biosynthesis by cholesterol 7α-hydroxylase (CYP7A1) is a major pathway for the removal of cholesterol into bile. A common promoter variant in *CYP7A1* (A-204C, rs3808607) is significantly associated with a poor response to atorvastatin (Kajinami et al., 2005; Kadam et al., 2016), which may be due to reduced gene expression (Pullinger et al., 2002). Patients with the GG or GT genotype of *CYP7A1* (rs3808607) may have a decreased response to atorvastatin compared to patients with the TT genotype (Kajinami et al., 2005; Kadam et al., 2016). In this study, subjects with GG or GT genotypes of *CYP7A1* (rs3808607) showed lower atorvastatin AUC values than those with the TT genotype (Supplementary Figure S2), which suggests that a decreased response to atorvastatin in subjects with the GG or GT genotype may be due to the lower atorvastatin AUC values in the GG or GT genotype compared to the TT genotype.

Our study also showed that the genetic variants of *APOA5* (rs33984246_G, rs3135507_T) and *CETP* (rs12708974_T) are associated with the AUC of atorvastatin. CETP plays an important role in cholesterol metabolism by bringing cholesterol esters into the liver and transferring triglycerides from LDL to HDL (Freeman et al., 1994). Genetic variants of *CETP* have been associated with cholesterol levels, response to statins, and clinical outcomes, such as myocardial infarction or stroke (Freeman et al., 1994; Kuivenhoven et al., 1998). Apolipoprotein A5 plays a key role in lipid metabolism, especially in the regulation of triglycerides. *APOA5* genetic variants are associated with hypertriglyceridemia (Martin-Campos et al., 2014). The *APOA5* variants (rs3135506) elevate triglyceride levels and shift the entire lipoprotein subclass distribution to atherosclerotic dyslipidemia in patients with high cardiovascular risk (Wang et al., 2008). Collectively, although the genetic variants of *CETP* might affect the efficacy of statins to reduce cardiovascular risk, and the variants of *APOA5* may be associated with hypertriglyceridemia, further studies are needed to elucidate the exact meaning of the findings in this study.

In this prospective study, we investigated the PK characteristics and lipid-related efficacy of a single high-dose atorvastatin administration in healthy Korean individuals. However, we acknowledge the limitations of our study. Because of the small sample size, our study may not have sufficient power to detect potentially relevant differences. Therefore, most associations could have been missed or underestimated. Moreover, a considerable number of statistically significant SNPs have not been previously studied. It will also be important to verify our results in prospective studies performed on a larger scale, with various ethnic populations, including other Asian subjects, because the nonreproducibility of results commonly undermines genetic association studies.

The strength of our study is that this is the first demonstration to estimate atorvastatin AUC based on the information of genetic variants associated with atorvastatin pharmacokinetics. Moreover, because this study utilized next-generation sequencing (NGS) technology for genomic analysis, it provides more comprehensive information than previous sequencing studies. Our findings may contribute to the basic data needed for therapeutic drug monitoring. Further studies on the correlation between the AUC or C_max_ of atorvastatin and the side effects of atorvastatin could support the value of further pharmacogenomic studies on inter-individual PK of atorvastatin.

## Conclusion

We demonstrated the PK parameters of atorvastatin and its active metabolites and analyzed the values 24 h after single high-dose statin administration in a healthy Korean population. The mean atorvastatin area under the concentration-time and maximum peak concentration was approximately two times higher than that reported in Caucasians, which is consistent with a previous report of increased statin sensitivity in Asian subjects. Furthermore, we also developed an atorvastatin AUC_0–24 h_ prediction model based on the eight genetic variants related to atorvastatin pharmacokinetics. This study suggests that genetic variants of *ABCB1*, *ABCG2*, *APOA5*, *CETP*, and *CYP7A1* contribute to the variability of atorvastatin exposure in the Korean population.

## Data Availability

The original contributions presented in the study are publicly available. This data can be found on NCBI SRA, BioProject: PRJNA791416 (https://www.ncbi.nlm.nih.gov/bioproject/PRJNA791416).
