# Supplementary material for: Pharmacokinetics and Genetic Factors of Atorvastatin in Healthy Korean Subjects
Source: Front Genet. 2022 May 19;13:836970. doi: 10.3389/fgene.2022.836970 (PMC9160745; doi:10.3389/fgene.2022.836970)
Supplement: Supplementary file 1 [file DataSheet1.pdf]

Table S1. Statin panel design information. 15 genes and 46 non-exon loci (56,432bp targeted region)

Candidates	Function	Full gene / SNV (Genomic coordinate. GRch37)	Reference
ABCA1	Cholesterol efflux pump	rs12003906 (chr9:107645477)	PharmGKB
ABCB1	Xenobiotic compounds transporter	Full gene (CDS+UTR)	Genet Med. 2014;16(11):810-9.
ABCC2	Anionic conjugates transporter	rs717620 (chr10:101542578)	PharmGKB
ABCG2	Xenobiotic compounds transporter	Full gene (CDS+UTR)	Genet Med. 2014;16(11):810-9.
ABCG2		rs1481012 (chr4:89039082)	PharmGKB
ABCG2		rs2199936 (chr4:89045331)	PharmGKB
ABCG8	ATP-binding cassette (ABC) transporter	rs11887534 (chr2:44066247)	PharmGKB
APOA5	Apolipoprotein A5	Full gene (CDS+UTR)	Pharmacogenomics. 2009;10(6):945-50.
APOA5		rs662799 (chr11:116663707)	PharmGKB
APOB	Apolipoprotein B	Full gene (CDS+UTR)	Genereviews: Familial Hypercholesterolemia
APOC1	Apolipoprotein C1	rs4420638 (chr19:45422946)	PharmGKB
APOE	Apolipoprotein E	Full gene (CDS+UTR)	PharmGKB
ATP2B1	Plasma membrane calcium ATPase	rs17381194 (chr12:90011095)	Atherosclerosis. 2011;218(2):451-6.
CETP	Transfer of cholesteryl ester from HDL to other lipoproteins	Full gene (CDS+UTR)	JAMA. 2017;318(10):947-956.
CETP		rs1532624 (chr16:57005479)	PharmGKB
CETP		rs708272 (chr16:56996288)	PharmGKB
COQ2	Redox carrier in the mitochondrial respiratory chain	Full gene (CDS+UTR)	PharmGKB, Neuromuscul Disord. 2014;24(1):4-15.
COQ2		rs4693075 (chr4:84192168)	PharmGKB
CRP	C-reactive protein	rs1205 (chr1:159682233)	PharmGKB
CXCL5	G-protein coupled receptor chemokine receptor	rs352046 (chr4:74864550)	PharmGKB
CYP2D6	Cytochrome P450 enzyme family 2 subfamily D member 6	Full gene (CDS+UTR)	Genet Med. 2014;16(11):810-9.
CYP2D6		rs28371725 (chr22:42523805)	Lowered CYP2D6 Activity
CYP3A4	Cytochrome P450 enzyme family 3 subfamily A member 4	Full gene (CDS+UTR)	Genet Med. 2014;16(11):810-9.
CYP3A4		rs2740574 (chr7:99382096)	Clin Chim Acta. 2012;413(3-4):495-501.

CYP3A4		rs35599367 (chr7:99366316)	Pharmacogenomics J. 2011;11(4):274-86.
CYP3A5	Cytochrome P450 enzyme family 3 subfamily A member 5	Full gene (CDS+UTR)	Genet Med. 2014;16(11):810-9.
CYP3A5		rs17161788 (chr7:99245909)	PharmGKB
CYP3A5		rs776746 (chr7:99270539)	PharmGKB
CYP7A1	Cytochrome P450 enzyme family 7 subfamily A member 1	rs3808607 (chr8:59412924)	PharmGKB
DMPK	Serine-threonine kinase	rs672348 (chr19:46284334)	Atherosclerosis. 2011;218(2):451-6.
FMO3	Drug-metabolizing enzymes	rs1736557 (chr1:171080080)	PharmGKB
GATM	Mitochondrial amidino transferase enzyme family	Full gene (CDS+UTR)	Genet Med. 2014;16(11):810-9.
HMGCR	HMG-CoA reductase	Full gene (CDS+UTR)	PharmGKB
HMGCR		rs17238540 (chr5:74655498)	PharmGKB
HMGCR		rs17244841 (chr5:74642855)	PharmGKB
HMGCR		rs17671591 (chr5:74615021)	PharmGKB
HTR3B	Ligand-gated ion channel receptor	rs2276307 (chr11:113803887)	PharmGKB
HTR7	Serotonin receptor	rs1935349 (chr10:92594343)	PharmGKB
KIF6	Involved in intracellular transport of protein	rs20455 (chr6:39325078)	PharmGKB
LDLR	Low density lipoprotein receptor	Full gene (CDS+UTR)	PharmGKB
LDLR		rs1433099 (chr19:11242658)	PharmGKB
LPA	Serine proteinase constitutes a lipoprotein(a)	rs10455872 (chr6:161010118)	PharmGKB
MYLIP	Cytoskeletal effector proteins linking actin-membrane protein	rs9370867 (chr6:16145325)	PharmGKB
PCSK9	Serine protease reduce LDL receptor	Full gene (CDS+UTR)	Genereviews: Familial Hypercholesterolemia
POR	Endoplasmic reticulum membrane oxidoreductase	rs1057868 (chr7:75615006)	PharmGKB
RYR2	Ryanodine receptor in cardiac muscle	rs2819742 (chr1:237990122)	PharmGKB
SCARB1	Plasma membrane receptor for HDL cholesterol	rs5888 (chr12:125284748)	PharmGKB
SLCO1B1	Liver-specific organic anion transmembrane transporter	Full gene (CDS+UTR)	Genet Med. 2014;16(11):810-9.
SLCO1B1		rs2900478 (chr12:21368797)	PharmGKB
SLCO1B1		rs4149036 (chr12:21327740)	PharmGKB

SLCO1B1		rs4149081 (chr12:21378021)	PharmGKB
TCF7L2	HMG box-containing transcription factor	rs11196205 (chr10:114807047)	BMC Med Genet. 2009;10:15.
TCF7L2		rs11196218 (chr10:114840494)	Meta Gene. 2014;2:332-41.
TCF7L2		rs11196251 (chr10:114926790)	dbSNP
TCF7L2		rs113102683 (chr10:114710163)	dbSNP
TCF7L2		rs12255372 (chr10:114808902)	Diabetes. 2006;55(9):2645-8. BMC Med Genet. 2009;10:15.
TCF7L2		rs574873054 (chr10:114710208)	dbSNP
TCF7L2		rs5787989 (chr10:114926210)	dbSNP
TCF7L2		rs77961654 (chr10:114925369)	dbSNP
TCF7L2		rs7901695 (chr10:114754088)	BMC Med Genet. 2009;10:15.
TCF7L2		rs7903146 (chr10:114758349)	BMC Med Genet. 2009;10:15.

Table S2. Genetic polymorphisms associated with pharmacokinetic parameters of atorvastatin

Gene	rs ID	Nucleotide change	MAF	AF global	AF EAS	AF EUR	aA Allele	Phenotype	Mean A/Ab	Mean A/B	Mean B/B	Beta	Unit	p-value	FDR p
ABCB1	rs1922242	NM_000927.4:c.2065-76T>A	0.300	0.427	0.292	0.429	A	$AUC_{0-24\ h}$	303.4	309.3	217.9	83.47	ng·h /mL	0.032	0.054
	rs3214119	NM_000927.4:c.-6-74delG	0.071	0.048	0.089	0.027	delG	$AUC_{0-24\ h}$	249.1	395.2	NA	141.50	ng·h /mL	0.015	0.028
	rs2235029	NM_001348945.2:c.1324-41T>G	0.014	0.002	0.022	0.000	G	$AUC_{0-24\ h}$	NA	121.1	274.4	-217.20	ng·h /mL	0.097	0.137
ABCG2	rs1564481	NM_004827.2:c.-19-99G>A	0.328	0.324	0.266	0.373	T	$AUC_{0-24\ h}$	167.2	248.6	315.7	-0.64	ng·h /mL	0.007	0.377
								CL/F	485.1	343.1	294.8	0.63	L/h	0.007	0.421
	rs182367277	NM_001257386.2:c.-260C>T	0.014	0.000	0.006	0.000	A	$AUC_{0-24\ h}$	.	714.5	256.9	2.15	ng·h/mL	0.029	0.377
								CL/F	.	107.8	344.0	-2.17	L/h	0.026	0.421
								C_{max}	.	223.0	84.8	2.19	ng/mL	0.026	0.616
								$T_{1/2}$	.	6.8	5.5	1.94	h	0.030	0.567
	rs2231153	NM_004827.2:c.1367+20G>A	0.743	0.849	0.783	0.960	T	C_{max}	82.3	109.2	93.5	-0.73	ng/mL	0.036	0.616
	rs2231162	NM_004827.2:c.1648-21T>C	0.857	0.931	0.797	0.963	G	C_{max}	82.3	109.2	93.5	-0.73	ng/mL	0.036	0.616
	rs3102038	NM_004827.2:c.532-2482C>T	0.343	0.355	0.266	0.373	A	$AUC_{0-24\ h}$	167.2	264.4	303.3	-0.56	ng·h/mL	0.021	0.377
								CL/F	485.1	331.0	304.6	0.55	L/h	0.022	0.421
	rs34262876	NM_004827.2:c.1647+65T>G	0.014	0.000	0.006	0.000	C	$T_{1/2}$	.	8.1	5.4	1.81	h	0.042	0.567
	rs759701118	NM_004827.2:c.1521C>T	0.014	0.000	0.000	0.000	A	$AUC_{0-24\ h}$	.	714.5	256.9	2.15	ng·h/mL	0.029	0.377
								CL/F	.	107.8	344.0	-2.17	L/h	0.026	0.421
								C_{max}	.	223.0	84.8	2.19	ng/mL	0.026	0.616
								$T_{1/2}$	.	6.8	5.5	1.94	h	0.030	0.567
APOA5	rs33984246	NM_052968.4:c.*394A>G	0.085	0.057	0.125	0.027	G	$AUC_{0-24\ h}$	-	381.3	231.4	1.05	ng·h/mL	0.005	0.399
								CL/F	-	228.7	374.9	-1.03	L/h	0.006	0.414
	rs45596738	NM_052968.4:c.*288_*289dup	0.085	0.057	0.123	0.027	dupTC	$AUC_{0-24\ h}$	-	381.3	231.4	1.05	ng·h/mL	0.005	0.399
								CL/F	-	228.7	374.9	-1.03	L/h	0.006	0.414
	rs34089864	NM_052968.4:c.*76G>A	0.065	0.039	0.124	0.025	A	$AUC_{0-24\ h}$	-	381.3	231.4	1.05	ng·h/mL	0.005	0.399
								CL/F	-	228.7	374.9	-1.03	L/h	0.006	0.414
	rs3135507	NM_052968.4:c.457C>T	0.082	0.051	0.119	0.030	T	$AUC_{0-24\ h}$	-	402.3	242.9	1.03	ng·h/mL	0.009	0.428
CEPT	rs12708974	NM_000078.2:c.658+257C>T	0.090	0.091	0.120	0.119	T	$AUC_{0-24\ h}$	-	188.7	298.1	-1.14	ng·h/mL	0.002	0.399
								C_{max}	-	59.7	98.8	-1.21	ng/mL	0.001	0.254
								CL/F	-	438.5	302.2	1.16	L/h	0.001	0.414
	rs1801706	NM_000078.2:c.*84G>A	0.155	0.168	0.090	0.174	A	C_{max}	-	60.5	95.8	-1.07	ng/mL	0.007	0.616
CYP7A1	rs1385203436	NG_007969.1:g.4896dup	0.085	0.001	0.000	0.000	GA	C_{max}	.	129.0	80.4	0.95	ng/mL	0.024	0.616
	rs3808607	NG_007969.1:g.4798C>T	0.571	0.530	0.558	0.582	T	$AUC_{0-24\ h}$	368.8	235.6	209.5	0.70	ng·h/mL	0.005	0.399
								CL/F	240.1	369.4	403.2	-0.68		0.006	0.414
								C_{max}	122.3	77.1	68.0	0.66	ng/mL	0.009	0.616

PCSK9	rs201349983	NM_174936.3:c.996+55_996+61del	0.993	0.842	0.787	0.896	G	CL/F	325.3	464.9	-	-1.50	L/h	0.010	0.455
	rs505151	NM_174936.3:c.2009G>A	0.899	0.942	0.947	0.962	A	$T_{1/2}$	5.3	7.0	-	-1.29	h	0.005	0.854
SLCO1B1	rs2900478	NM_006446.4:c.1498-1256T>A	0.090	0.142	0.105	0.185	A	AT C_{max}	-	109.5	81.5	1.03	ng/mL	0.006	0.616

AF, from gnomAD genome database [a]A, variant allele; [b]A/A, variant homozygotes; A/B, heterozygotes; B/B, Reference homozygotes; Metabolites, p-OH atorvastatin and o-OH atorvastatin

Table S3. Genetic polymorphisms found only in the two cases who had extreme pharmacokinetic phenotypes of ATV and its metabolites

Case	Gene	SNP ID	Nucleotide change	MAF	A [a] Allele	Parameter	Mean			Beta	Unit	*p*-value	FDR *p*
							A/A[b]	A/B	B/B				
Participant 16	*ABCB1*	chr7:87135155	NM_000927.4:c.3636+58T>C	-	C	ATV AUC_{∞}	-	741.8	265.8	2.166	ng·h/mL	0.026	0.569
						AT C_{max}	-	223.0	84.78	2.186	ng/mL	0.003	0.616
	ABCG2	rs182367277	NM_001257386.1:c.-260G>A	0.002	A	ATV AUC_{∞}	-	741.8	265.8	2.166	ng·h/mL	0.026	0.569
						ATV C_{max}	-	223.0	84.78	2.186	ng/mL	0.003	0.616
		rs759701118	NM_004827.2:c.1521G>A	< 0.001	A	ATV AUC_{∞}	-	741.8	265.8	2.166	ng·h/mL	0.026	0.569
						ATV C_{max}	-	223.0	84.78	2.186	ng/mL	0.003	0.616
	CYP3A5	rs139485693	NM_000777.4:c.1026+176G>C	0.002	C	ATV AUC_{∞}	-	741.8	265.8	2.166	ng·h/mL	0.026	0.569
						ATV C_{max}	-	223.0	84.78	2.186	ng/mL	0.003	0.616
	KIF6	rs78761242	NM_145027.4:c.2180+19C>T	0.003	T	ATV AUC_{∞}	-	741.8	265.8	2.166	ng·h/mL	0.026	0.569
						ATV C_{max}	-	223.0	84.8	2.186	ng/mL	0.003	0.616
Participant 11	*CEPT*	rs12691052	NM_000078.2:c.930+90G>A	0.044	A	Metabolites C_{max}	113.2	58.5	48.9	0.295	ng/mL	0.029	0.557
		rs891144	NM_000078.2:c.982-67C>T	0.111	T	Metabolites C_{max}	113.2	58.5	48.9	0.295	ng/mL	0.029	0.557
	HMGCR	rs3761741	NM_000859.3:c.-17T>G	0.016	G	Metabolites AUC0-24 h	-	418.0	268.4	2.244	ng·h/mL	0.042	0.845
	CYP2D6	rs138098289	NM_000106.5:c.353-79C>T	< 0.001	T	Metabolites AUC0-24 h	-	418.0	268.4	2.244	ng·h/mL	0.042	0.845
	PCSK9	chr1:55527265	NM_174936.3:c.1863+36T>C	-	-	Metabolites AUC0-24 h	-	418.0	268.4	2.244	ng·h/mL	0.042	0.845

MAF, from 1000 genome database; [a]A, minor allele; [b]A/A, minor homozygotes; A/B, heterozygotes; B/B, major homozygotes.

Abbreviations: ATV, atorvastatin; AUC_{∞}, area under the plasma concentration curve from hour 0 to infinity; C_{max}, maximum plasma drug concentration; FDR, false discovery rate; MAF, minor allele frequency; SNP, single nucleotide polymorphism.

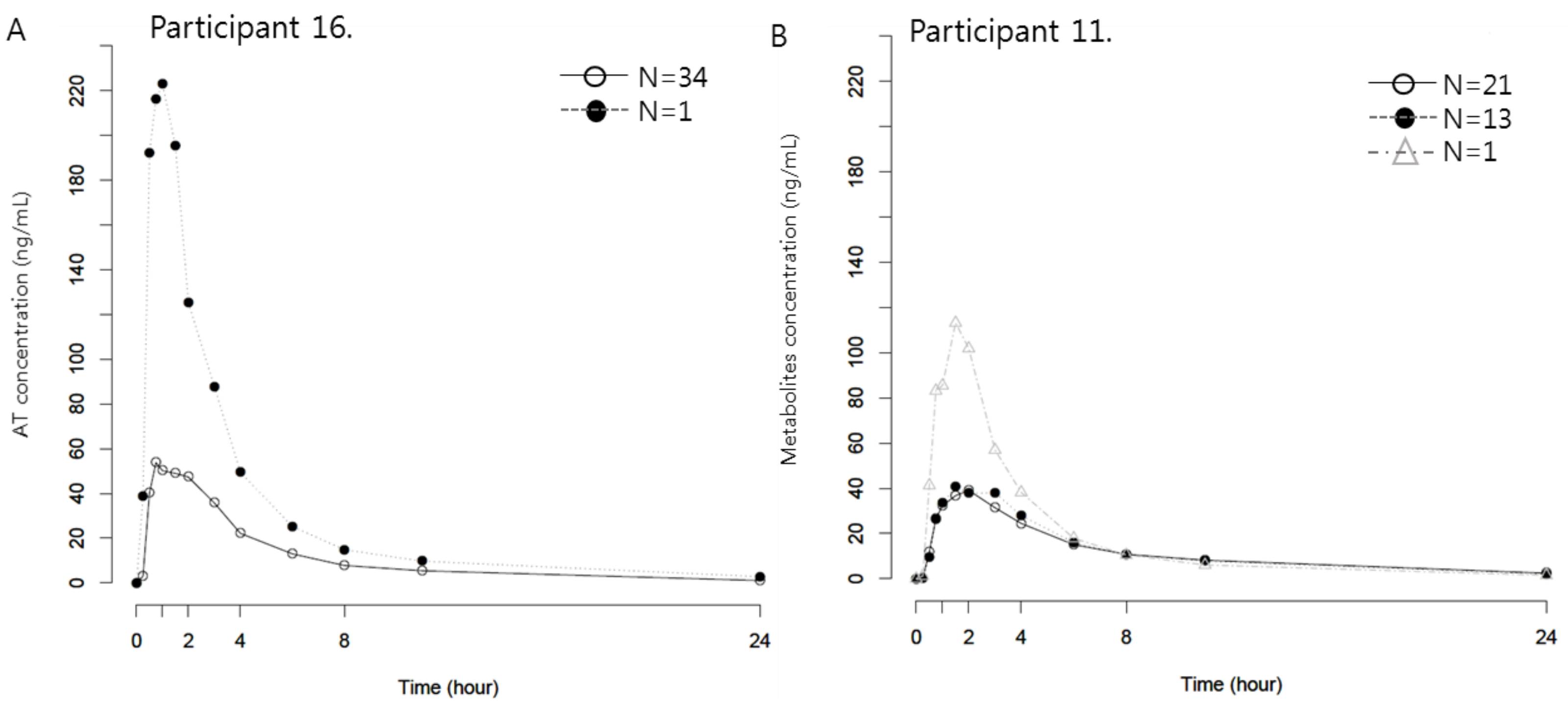

Figure S1. Plasma concentration-time plots of atorvastatin and its metabolites after receiving a single dose of 80 mg atorvastatin. Pharmacokinetic parameters of atorvastatin or its metabolites in these individuals were more than twice as much as the average of those of the other participants.

Notes: (A) Solid circles represent mean plasma atorvastatin concentrations of participant 16, who had one variant allele (heterozygote) for *ABCB1* c.3636+58T>C, *ABCG2* c.-260G>A, *ABCG2* c.1521G>A, *CYP3A5* 1026+176G>C, and *KIF*6 2180+19C>T; and the hollow circles indicate the concentrations of the other 34 subjects who had no variant alleles of those polymorphisms (major homozygotes). (B) Hollow triangles represent mean plasma metabolites concentrations of participant 11, who had two variant alleles (minor homozygote) for *CEPT* c.930+90G>A, *CEPT* c.982-67C>T; the solid circles represent the concentrations of 13 subjects who had one variant allele (heterozygotes); and the hollow circles indicate the concentrations of the other 21 subjects who had no variant alleles (major homozygotes) for the above mentioned polymorphisms.

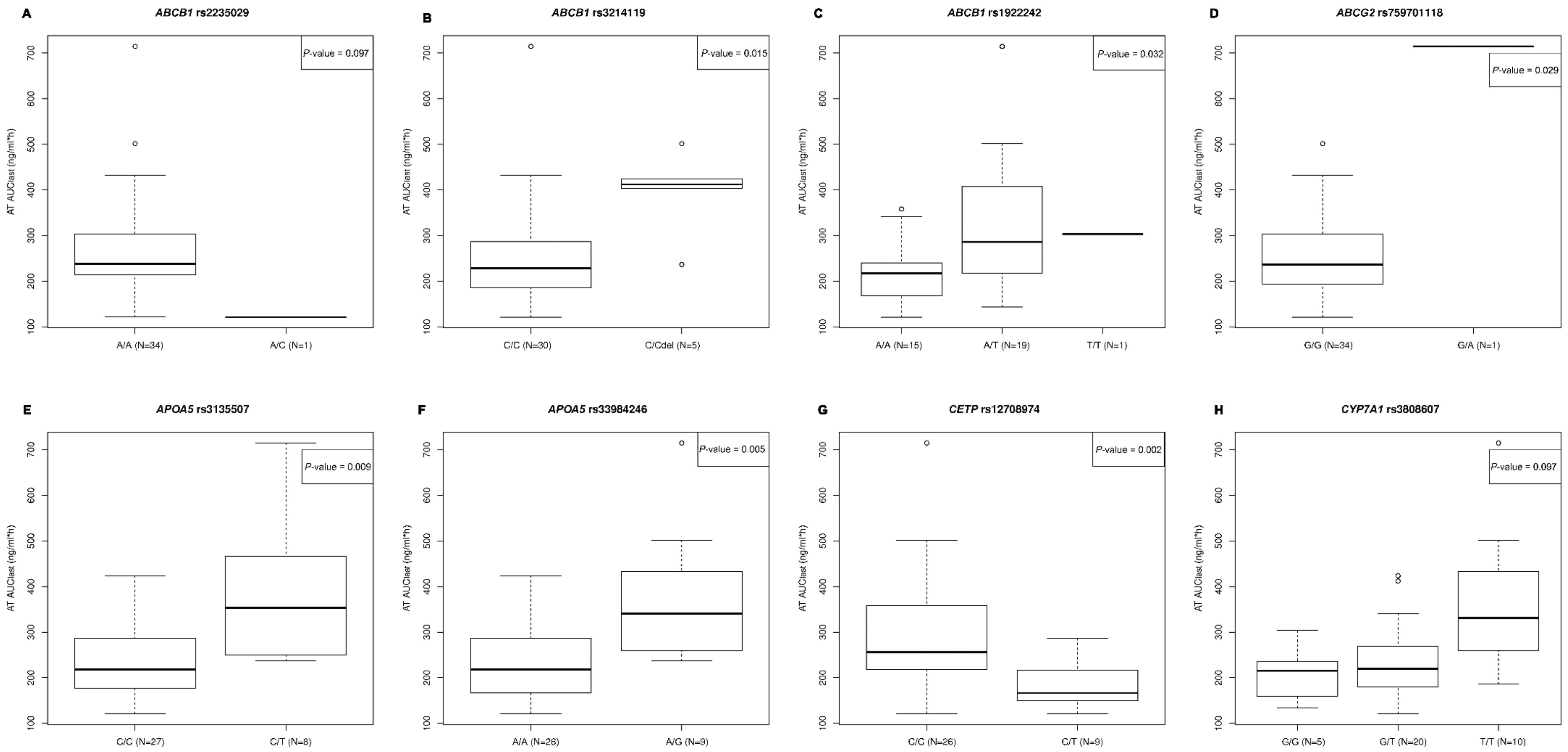

Figure S2. ATV AUC differences according to the 8 genetic variants, which contribute the ATV AUC in the multivariate linear regression analysis (*ABCB1* rs2235029_C, rs3214119_T, rs1922242_T; *ABCG2* rs759701118_A; *APOA5* rs33984246_G, rs3135507_T; *CETP* rs12708974_T; *CYP7A1* rs3808607_T). No polymorphisms exceed the FDR significance threshold *p*-values < 0.05.

Abbreviations: ATV, atorvastatin; AUC0-24 h, area under the plasma concentration curve form hour 0 to observation, C_{max}, maximum plasma drug concentration; Vz/F, apparent volume of distribution during terminal phase after non-intravenous administration.
